# Supplementary material for: Performance of deep-learning-based approaches to improve polygenic scores
Source: Nat Commun. 2025 Jun 2;16:5122. doi: 10.1038/s41467-025-60056-1 (PMC12130321; doi:10.1038/s41467-025-60056-1)
Supplement: Supplementary file 1 — Supplementary Information [file 41467_2025_60056_MOESM1_ESM.pdf]

Supplementary material for "Performance of deep-learning based approaches to improve polygenic scores"

Supplementary Note 1: details of phenotype simulation involving epistasis

Simulating phenotypes made up from nonlinear genetic effects involves additional parameters and difficulties, relative to phenotypes due to purely additive effects. There are two parameters that need to be considered for phenotypes involving epistatic effects. The first parameter is the number of SNPs that have a causal effect on the phenotype *c*, which would be the only parameter needed for simulating additive phenotypes. However, there is also a second parameter *v*, which controls the number of interactions made up from the *c* SNPs. Preliminary analyses indicated that while *v* had an impact on the overall accuracy, it did not seem to influence the preference between the linear and nonlinear methods, therefore we set *v* = *c* (i.e. all causal SNPs have an interaction).

To reduce the search space, only two phenotypes were simulated, one where the phenotype was due to entirely four-way interactions and another one entirely due to additive effects. Thus, the raw genetic values *GV* for individual *j* were calculated as

$$GV^j = \sum_i^v X_i^j \beta_i \quad ; \quad \beta_i \sim N(0,1), \tag{1}$$

where β_i are the SNP (interaction) effects drawn from a standard normal distribution, and X_i^j denotes the *v* randomly selected combinations of SNPs selected to be causal. Thus, X_i^j was defined as

$$X_i^j = \prod_d^{D_i} SNP_{dj}, \tag{2}$$

where SNP_{dj} is the genotype count for the *d*th SNP in the *i*th fourth-order interaction.

To simulate phenotypes with an additive genetic architecture with a predefined heritability, the proportion of phenotypic variance due to genetic variance (h^2), the final phenotype (y_{sim}) is the sum of the genetic (**g**) and noise (**e**) components as

$$\boldsymbol{y_{sim}} = \boldsymbol{g} + \boldsymbol{e}, \quad (3)$$

where both **g** and **e** are scaled in proportion to the desired h^2. The noise component is drawn from a standard normal distribution, with zero mean and a variance of $1 - h^2$

$$e \sim N(0, \sqrt{1 - h^2}). \quad (4)$$

Thus, the noise contributes all the remaining variance not due to h^2. The scaled genetic value (***g***) is in turn defined as

$$\boldsymbol{g} = \boldsymbol{GV} * s, \quad (5)$$

where *s* is a scaling factor given by

$$s = \sqrt{\frac{h^2}{var(\boldsymbol{GV})}}, \quad (6)$$

where $var(\boldsymbol{GV})$ denotes the sample variance of the genetic values. The above would generate a simulated phenotype, arising from additive genetic effects, with a pre-specified level of h^2. However, to generate phenotypes due to a genetic architecture from nonlinear effects, the above formula was modified by adjusting the scaling factor *s* to be proportionate to the desired apparent additive effect of SNPs. This was accomplished by fitting a multiple linear regression model, and regressing the individual genetic values ($\boldsymbol{GV}$) on the genotype matrix as

$$\boldsymbol{GV} = \mathbf{X}'\boldsymbol{\beta'} + \boldsymbol{\epsilon}, \quad (7)$$

where $\mathbf{X}'$, $\boldsymbol{\beta'}$ and $\boldsymbol{\epsilon}$ denote the genotype matrix of the individual SNPs involved in interactions, the interaction coefficients and a random noise term, respectively. The fitted values from this model ($\widehat{\boldsymbol{GV}}$), approximate the genetic values due to the apparent main effects of the SNPs involved in interactions. By using this new $\widehat{\boldsymbol{GV}}$ a new scaling factor may be derived by

$$s' = \sqrt{\frac{h^2}{var(\widehat{\boldsymbol{GV}})}}. \quad (8)$$

From this point onward, with the exception of using this new scaling factor *s'*, the rest of the simulation steps were identical to the phenotypes due to purely additive effects.

Supplementary Note 2: parameter & model selection for NNs for PGS generation

In this work, the performance of NNs was taken as the better of two MLPs, both with three hidden layers: a 'large' one with 100, 50 and 25, and another 'small' one with 24, 12 and 6 neurons in each hidden layer, respectively. The architecture of these models was chosen based on our prior experience working with NNs for generating PGS. During the first author's PhD work a more extensive model search was performed, including evaluating the performance of convolution layers, number of hidden layers between 1 and 20, and the number of neurons in the first layer varying between 100 and 4000. There, it was found that smaller models with fewer layers tended to outperform larger models and that convolutional layers had a negative effect on performance. More details and empirical tests can be found in the studies by Bellot et al. (2018), Kelemen et al. (2021) and Xu et al. (2022)) [1–3].

The phenomenon of smaller, simpler NNs performing better than larger, more complex ones is consistent with the expected result when the amount of non-linearity is low/non-existent, as that is when the NN model gets closest to a basic linear regression model (one layer with one neuron). Convolutional NNs (CNNs) are ubiquitous in image and DNA sequence classification tasks where they routinely outperform MLPs. We attribute the lack of success of CNNs in PGS generation to the differences between the nature of image or DNA sequence data and individual-level genetic data. CNNs may outperform MLPs if the input data follows a regular spatial structure. Image labels are predicted from pixel data, where convolutional layers may learn reusable features. For example, a 3x3 filter in an image classification task may represent an edge. Similarly, for a sequence classifier NN, a motif pattern (example: *'GCA'*) may represent a (partial) transcription factor binding site, both of which are reusable features that are likely to carry the same meaning in other areas of the input. However, convolutions do not fit individual-level genetic data well. This is because SNP data represents deltas from a reference genome with an arbitrary distance between the nucleotides. Given that sequence context is absent in SNP data, reusable filters are unlikely to be learned here. To illustrate, consider an example pattern of three SNPs *'021'*. The same three values are unlikely to carry the same meaning elsewhere, therefore, reusing this pattern may not benefit the model.

Supplementary Tables

83 Binary or numeric covariates	115 Multi-level factor covariates
Age at survey (yrs), Sex, Time in education (yrs), Smoking status, Smoking amount: unknown type (pack years), Smoking amount: combined (pack years), Smoking amount: combined (cigarettes/day), Alcohol status, Alcohol amount: combined, Alcohol frequency: combined (days/week), History of diabetes, Anti-diabetic drug status, HRT drug status, Anti-hypertensives drug status, Lipid-lowering unspecified drug status, SBP (mmHg), DBP (mmHg), Height (cm), Weight (kg), BMI (kg/m2), Waist (cm), Hip (cm), Waist/hip ratio, Waist/height ratio, Total cholesterol (mmol/l), HDL-C (mmol/l), Non-HDL-C (mmol/l), LDL-C (mmol/l), Triglycerides (mmol/l), Apolipoprotein A1 (g/l), Apolipoprotein B (g/l), Lp(a) (mg/dl), Haematocrit (%), Haemoglobin (g/l), White cell count (x10^9/l), Forced Expiratory Volume (l/1min), CRP (mg/l), Total protein (g/l), Albumin (g/l), Creatinine (µmol/l), Glucose (mmol/l), HbA1c (%), Eosinophils (%), Urine Creatinine (mmol/l), Urine Potassium (mmol/l), Urine Microalbumin (mg/L), Urine Sodium (mmol/l), Cystatin-c (mg/l), Urine Creatinine upper or lower bound, Urine Potassium upper or lower bound, Urine Microalbumin upper or lower bound, Urine Sodium upper or lower bound, Alkaline Phosphatase (iu/l), Alanine Transaminase (also called GPT) (iu/l), Aspartate Aminotransferase (also called GOT or SGOT) (iu/l), Basophils (%), Calcium (mmol/l), Direct Bilirubin (µmol/l), Gamma-Glutamyl Transferase (iu/l), Hand grip strength left (kg), Hand grip strength right (kg), Insulin-like Growth Factor 1 (nmol/l), Lymphocytes (%), Monocytes (%), Neutrophils (%), Oestradiol (pmol/l), PEF, Phosphate (mmom/L), Platelet Estimate (10^9/l), Pulse (per min), Red blood cell count (10^12/l), Rheumatoid factor (iu/ml), Sex Hormone-Binding Globulin (nmol/l), Total bilirubin (µmol/l), Testosterone (nmol/l), Urea (mmol/l), Uric acid (µmol/l), Vital capacity (l), Vitamin D (25 dihydroxy-vitamin D) (nmol/L), History of chronic obstructive pulmonary disease (COPD), income: annual amount (cont), income: annual amount (cont)	Race, Nationality, Level of education reached, Occupation: job, Smoking status: cigarettes, Smoking status: pipes & cigars, Smoking status: unknown type, Smoking status: combined, Alcohol status: combined, History of CHD, History of MI, History of angina, History of other HD, History of stroke, History of ischaemic stroke, History of haemorrhagic stroke, History of TIA, History of PVD, History of diabetes, History of hypertension, History of cardiovascular surgery, History of coronary revascularisation (surgery), History of CABG (coronary artery bypass graft), History of PTCA (percutaneous transluminal angioplasty), History of vascular surgery, History of neoplasm, Drug status: anti-diabetics, Drug status: HRT, Drug status: anti-hypertensives, Drug status: lipid-lowering unspecified, Family history of CHD - parents, Family history of diabetes - parents, Family history of stroke - parents, Status, All cardiovascular, All cardiovascular (fatal), All cardiovascular (non-fatal), All CHD, All CHD (non-fatal), All CHD (fatal), All CHD plus all cerebrovascular, All CHD plus all cerebrovascular (fatal), All CHD plus all cerebrovascular (non-fatal), Myocardial infarction, Myocardial infarction (fatal), Myocardial infarction (non-fatal), CHD death and non-fatal MI, All cerebrovascular, All cerebrovascular (non-fatal), All cerebrovascular (fatal), Ischaemic stroke, Ischaemic stroke (non-fatal), Ischaemic stroke (fatal), Haemorrhagic stroke, Haemorrhagic stroke (fatal), Haemorrhagic stroke (non-fatal), Subarachnoid haemorrhage, Subarachnoid haemorrhage (fatal), Subarachnoid haemorrhage (non-fatal), Unclassified stroke, Unclassified stroke (fatal), Unclassified stroke (non-fatal), All unknown cause (fatal), All non-cardiovascular (fatal), All tumour (fatal), Digestive related cancer (fatal), Lung cancer (fatal), Genitourinary related cancer (fatal), Breast cancer (fatal), All non-tumour non-cardiovascular (fatal), External (violence/suicide/trauma) (fatal), Infectious/bacterial/parasitic (except hepatitis) (fatal), Mental disorder (fatal), Nervous system disorder (fatal), Liver disease (fatal), Respiratory system disease (fatal), Digestive system disease (except liver) (fatal), Renal disease (fatal), COPD and related conditions (fatal), COVID-19 (fatal), Heart failure (fatal), Status (all-cause mortality), All cardiovascular (all-cause mortality), All CHD (all-cause mortality), All CHD plus all cerebrovascular (all-cause mortality), Myocardial infarction (all-cause mortality), All cerebrovascular (all-cause mortality), Ischaemic stroke (all-cause mortality), Haemorrhagic stroke (all-cause mortality), Subarachnoid haemorrhage (all-cause mortality), Unclassified stroke (all-cause mortality), All unknown cause (all-cause mortality), All non-cardiovascular (all-cause mortality), All tumour (all-cause mortality), Digestive related cancer (all-cause mortality), Lung cancer (all-cause mortality), Genitourinary related cancer (all-cause mortality), Breast cancer (all-cause mortality), All non-tumour non-cardiovascular (all-cause mortality), External (violence/suicide/trauma) (all-cause mortality), Infectious/bacterial/parasitic (except hepatitis) (all-cause mortality), Mental disorder (all-cause mortality), Nervous system disorder (all-cause mortality), Liver disease (all-cause mortality), Respiratory system disease (all-cause mortality), Digestive system disease (except liver) (all-cause mortality), Renal disease (all-cause mortality), COPD and related conditions (all-cause mortality), COVID-19 (all-cause mortality), Heart failure (all-cause mortality), History of heart failure, History of respiratory system disease, Drug status: Birth Control Pill, Drug status: vitamin D

Supplementary Table 1 | Full list of covariates used in the real data analyses.

Target phenotype	Exclusion list
cancer	Breast cancer (all-cause mortality), Digestive related cancer (all-cause mortality), Genitourinary related cancer (all-cause mortality), All tumour (fatal), Digestive related cancer (fatal), Lung cancer (fatal), Genitourinary related cancer (fatal), Breast cancer (fatal), Lung cancer (all-cause mortality)
hypertension	All cardiovascular, All cardiovascular (fatal), All cardiovascular (non-fatal), History of other HD, Drug status: lipid-lowering unspecified, Lipid-lowering unspecified drug status, History of hypertension, Drug status: anti-hypertensives, Anti-hypertensives drug status
stroke	All cardiovascular, All cardiovascular (fatal), All cardiovascular (non-fatal), History of other HD, Drug status: lipid-lowering unspecified, Lipid-lowering unspecified drug status, History of stroke, History of ischaemic stroke, History of haemorrhagic stroke, History of TIA, Ischaemic stroke, Ischaemic stroke (non-fatal), Ischaemic stroke (fatal), Haemorrhagic stroke, Haemorrhagic stroke (fatal), Haemorrhagic stroke (non-fatal), Unclassified stroke, Unclassified stroke (fatal), Unclassified stroke (non-fatal), Ischaemic stroke (all-cause mortality), Haemorrhagic stroke (all-cause mortality)
cvd	All cardiovascular, All cardiovascular (fatal), All cardiovascular (non-fatal), History of other HD, Drug status: lipid-lowering unspecified, Lipid-lowering unspecified drug status
diabetes	History of diabetes, History of diabetes, Drug status: anti-diabetics
alcohol	Alcohol status: combined, Alcohol status, Alcohol amount: combined, Alcohol frequency: combined (days/week)
smoking	Smoking status: cigarettes, Smoking status: pipes & cigars, Smoking status: unknown type, Smoking status: combined, Smoking status, Smoking amount: cigarettes (pack years), Smoking amount: pipes & cigars (pack years), Smoking amount: unknown type (pack years), Smoking amount: combined (pack years), Smoking amount: combined (cigarettes/day)
height	Waist/height ratio, Height (cm), BMI (kg/m2)
glucose	Glucose (mmol/l)
hba1c	HbA1c (%)

Supplementary Table 2 | List of excluded covariates for specific target phenotypes.

trait	EFO	category	Internally curated	ICD10	ICD9	field	code	Sex
Hodgkin's disease	EFO_0000183	cancer		C81	201	20001	1052	all
Breast cancer (female)	EFO_0000305	cancer		C50		20001	1002	female
Ischemic stroke	EFO_0000712	Cardio-metabolic		I63		20002	1081	all
Melanomas of skin (diagnosis or history)	EFO_0000756	cancer		C43	172	20001	1059	all
Prostate cancer	EFO_0001663	cancer		C61	185	20001	1044	male
Thyroid cancer	EFO_0002892	cancer		C73	193	20001	1065	all
Cancer of tongue	EFO_0003871	cancer		C01, C02	141	20001	1011	all
Basal cell carcinoma	EFO_0004193	cancer		C44		20001	1061	all
Venous thromboembolism	EFO_0004286	Cardio-metabolic		I80	451	20002	1068	all
Malignant neoplasm of testis	EFO_0005088	cancer		C62	186	20001	1045	male
Cancer of mouth	EFO_0005570	cancer		C04, C06	144, 145	20001	1004	all
Skin cancer	EFO_0009259	cancer		C43	172	20001	1059	all
Cancer of larynx	EFO_1000354	cancer		C32	161	20001	1006	all
Malignant neoplasm of rectum, rectosigmoid junction, and anus	EFO_1000657	cancer		C20, C21	154	20001	1021, 1023	all

Colon cancer	EFO_1001950	cancer		C18	153	20001	1022	all
Cancer within the respiratory system	MONDO_0000376	cancer		C30-C39	165	20001	1084	all
Cancer of bronchus; lung	MONDO_0001407	cancer		C34	162	20001	1001	all
Cancer of brain	MONDO_0001657	cancer		C71	191	20001	1032	all
Major Depressive Disorder	MONDO_0002009	behavioural		F33		20002	1286	all
Cancer of eye	MONDO_0002236	cancer		C69	190	20001	1030	all
Carcinoma in situ of skin	MONDO_0004641	cancer		D04	232	20001	1003	all
Cancer of bladder	MONDO_0004986	cancer		C67	188	20001	1035	all
type 2 diabetes	MONDO_0005148	Cardio-metabolic		E11		20002	1223	all
Colorectal cancer	MONDO_0005575	cancer		C18	153	20001	1020	all
Cancer of esophagus	MONDO_0007576	cancer		C15	150	20001	1017	all
Height	EFO_0004339	biometric	ht					all
Fasting glucose	EFO_0004465	Cardio-metabolic	glucose1					all
Glycated haemoglobin levels (HbA1c)	EFO_0004541	Cardio-metabolic	hba1c					all

Supplementary Table 3 | List of all 28 phenotypes and their definitions.

nonlinear component	formula
any data (**X**)	$\Delta = NN_{nonlinear}(\mathbf{X}) - NN_{linear}(\mathbf{X})$
genuine epistasis	$\Delta(SNP{*}PRS) - \Delta(SNP)$
genome-wide GxE	$\Delta(SNP{*}\mathrm{PRS}, \mathrm{Covs}) - \Delta(SNP{*}PRS) - \Delta(Covs)$
per-SNP GxE	$\Delta(SNP{*}PRS, \mathrm{Covs}) - \Delta(SNP{*}PRS) - \Delta(\mathrm{Covs}) - \Delta(PRS_{indi}, \mathrm{Covs})$

Supplementary Table 4 | Formulae used to infer the various components of the overall nonlinear effect. *NN* is the neural-network function. **X** refers to any data that may be used as input to the NN. ***Δ*** is the difference between a nonlinear and linear NN model. *SNP* refers to SNP allele dosage data. *SNP*PRS* refers to SNP allele dosage data premultiplied by per-SNP PRS weights. *Covs* are the environmental covariates. PRS_{indi} refers to individual-level PRS profile scores.

EFO	linear	nonlinear
EFO_0000183	1.631	1.747(s)
EFO_0000305	1.485	0.956
EFO_0000712	0.362(s)	0.421(s)
EFO_0000756	1.341(s)	1.056
EFO_0001663	0.87	0.851
EFO_0002892	0.921(s)	0.515
EFO_0003871	2.03(s)	2.107(s)
EFO_0004193	1.216(s)	1.157
EFO_0004286	0.595	0.945
EFO_0005088	1.083(s)	0.951
EFO_0005570	0.388	0.679(s)
EFO_0009259	1.557(s)	1.305(s)
EFO_1000354	0.504	1.11
EFO_1001950	0.859	0.838(s)
MONDO_0000376	0.663	0.774(s)
MONDO_0001407	0.459(s)	0.573
MONDO_0001657	-0.472	0.859
MONDO_0002009	0.386(s)	0.23
MONDO_0002236	1.669	1.716
MONDO_0004641	0.61(s)	0.551
MONDO_0004986	1.958(s)	1.562(s)
MONDO_0005148	0.484(s)	0.586
MONDO_0005575	0.748(s)	0.868
MONDO_0007576	0.888(s)	0.932(s)
EFO_0004339	0.955(s)	0.96
EFO_0004465	0.733(s)	0.838
EFO_0004541	0.954(s)	0.983

Supplementary Table 5 | Per phenotype breakdown of results for the SNP-dosage weighting real data scenario. The linear column shows the linear NN and the nonlinear column shows the nonlinear NN model performance on the test set. (s) indicates if the performance evaluated originated from the small model ([24, 12, 6] neurons) or the large model ([100, 50,

25] neurons), a choice made based on the validation set performance. Units are expressed as a fraction relative to the additive baseline, which was the PGS Catalog PGS.

pheno	linear	nonlinear
EFO_0000305	1.006	1.047
EFO_0000712	0.06(s)	0.06
EFO_0000756	1.226(s)	1.242
EFO_0001663	0.908	0.863
EFO_0002892	2.13	2.097
EFO_0004193	0.952(s)	0.951
EFO_0004286	0.869	-0.099
EFO_0009259	0.301	1.257
EFO_1001950	0.943(s)	0.935(s)
MONDO_0000376	0.98(s)	0.998
MONDO_0001407	-0.211(s)	-0.339(s)
MONDO_0001657	3.416	3.845
MONDO_0002009	0.914	0.949
MONDO_0004641	-0.066(s)	0.705
MONDO_0004986	-0.285	-0.369
MONDO_0005148	0.982	0.985
MONDO_0005575	1.216	1.222
MONDO_0007576	0.703	0.703(s)
EFO_0004339	0.971(s)	0.993
EFO_0004465	0.349(s)	0.203
EFO_0004541	0.619(s)	0.533

Supplementary Table 6 | Per phenotype breakdown of results for the SNP+Covariate real data scenario. The linear column shows the linear NN and the nonlinear column shows the nonlinear NN model performance on the test set. (s) indicates if the performance evaluated originated from the small model ([24, 12, 6] neurons) or the large model ([100, 50, 25] neurons), a choice made based on the validation set performance. Units are expressed as a fraction relative to the additive baseline, which was a multiple regression model that included the same covariates plus the PGS Catalog PGS.

	Typical NN task	Phenotype prediction
**Task challenge**	problem complexity	signal recovery
**Sample size**	large	small
**noise**	low	high
**spatially structured data**	yes	no

Supplementary Table 7 | Summary of differences between typical tasks where NNs excel at and phenotype prediction.

References

1. Kelemen, M. Modelling human complex traits with regression and neural-network based methods. (2021) doi:10.17863/CAM.72055.
2. Bellot, P., de los Campos, G. & Pérez-Enciso, M. Can Deep Learning Improve Genomic Prediction of Complex Human Traits? *Genetics* **210**, 809 (2018).
3. Xu, Y. *et al.* Machine learning optimized polygenic scores for blood cell traits identify sex-specific trajectories and genetic correlations with disease. *Cell Genom.* **2**, None (2022).
