# Supplementary material for: Performance of deep-learning-based approaches to improve polygenic scores
Source: Nat Commun. 2025 Jun 2;16:5122. doi: 10.1038/s41467-025-60056-1 (PMC12130321; doi:10.1038/s41467-025-60056-1)
Supplement: Supplementary file 2 — Reporting Summary [file 41467_2025_60056_MOESM2_ESM.pdf]

nature portfolio

Corresponding author(s): Martin Kelemen

Last updated by author(s): May 13, 2025

Reporting Summary

Nature Portfolio wishes to improve the reproducibility of the work that we publish. This form provides structure for consistency and transparency in reporting. For further information on Nature Portfolio policies, see our Editorial Policies and the Editorial Policy Checklist.

Please do not complete any field with "not applicable" or n/a. Refer to the help text for what text to use if an item is not relevant to your study. For final submission: please carefully check your responses for accuracy; you will not be able to make changes later.

Statistics

For all statistical analyses, confirm that the following items are present in the figure legend, table legend, main text, or Methods section.

n/a	Confirmed	
☐	☒	The exact sample size (n) for each experimental group/condition, given as a discrete number and unit of measurement
☒	☐	A statement on whether measurements were taken from distinct samples or whether the same sample was measured repeatedly
☐	☒	The statistical test(s) used AND whether they are one- or two-sided<br>*Only common tests should be described solely by name; describe more complex techniques in the Methods section.*
☐	☒	A description of all covariates tested
☒	☐	A description of any assumptions or corrections, such as tests of normality and adjustment for multiple comparisons
☐	☒	A full description of the statistical parameters including central tendency (e.g. means) or other basic estimates (e.g. regression coefficient) AND variation (e.g. standard deviation) or associated estimates of uncertainty (e.g. confidence intervals)
☐	☒	For null hypothesis testing, the test statistic (e.g. F, t, r) with confidence intervals, effect sizes, degrees of freedom and P value noted<br>*Give P values as exact values whenever suitable.*
☒	☐	For Bayesian analysis, information on the choice of priors and Markov chain Monte Carlo settings
☒	☐	For hierarchical and complex designs, identification of the appropriate level for tests and full reporting of outcomes
☒	☐	Estimates of effect sizes (e.g. Cohen's d, Pearson's r), indicating how they were calculated

Our web collection on statistics for biologists contains articles on many of the points above.

Software and code

Policy information about availability of computer code

Data collection	No new data were collected for this study, i.e. there is no relevant software/code relating to data collection.
Data analysis	Code availability<br>Software programs used in this study are all publicly available; PLINK2 and PLINK v1.9 can be downloaded from Christopher Chang's website [https://www.cog-genomics.org/plink/], PyTorch (v1.9.0+cu111) from the PyTorch website [https://pytorch.org/], R (v4.3.1) from the CRAN website [https://cran.r-project.org/] and Rstudio from the posit website [https://posit.co/products/open-source/rstudio/]. Code to perform all analyses reported in this manuscript is available at GitHub [github.com/mkelcb/dl-prs-paper] and Zenodo [https://doi.org/10.5281/zenodo.15324037].

For manuscripts utilizing custom algorithms or software that are central to the research but not yet described in published literature, software must be made available to editors and reviewers. We strongly encourage code deposition in a community repository (e.g. GitHub). See the Nature Portfolio guidelines for submitting code & software for further information.

Data

Policy information about availability of data

All manuscripts must include a data availability statement. This statement should provide the following information, where applicable:

- Accession codes, unique identifiers, or web links for publicly available datasets
- A description of any restrictions on data availability
- For clinical datasets or third party data, please ensure that the statement adheres to our policy

Data Availability
This research has been conducted using the UK Biobank Resource under Application Number 7439. Data access policies [http://www.ukbiobank.ac.uk/register-apply/] and a description of the genetic data [http://www.ukbiobank.ac.uk/scientists-3/genetic-data/] are available from the UK Biobank website. The Research Analysis Platform is open to researchers who are listed as collaborators on UK Biobank approved access applications. Data and scripts to reproduce figures and tables are provided in the Source data provided with this paper. Polygenic score data from the PGS Catalog is publicly available from https://www.pgscatalog.org/.
For the purpose of open access, the author has applied a Creative Commons Attribution (CC BY) licence to any Author Accepted Manuscript version arising from this submission.

Research involving human participants, their data, or biological material

Policy information about studies with human participants or human data. See also policy information about sex, gender (identity/presentation), and sexual orientation and race, ethnicity and racism.

Reporting on sex and gender	We only use the term 'sex' throughout the manuscript as we were interested in biological sex. We only kept individuals in our analysis whose recorded gender and inferred genotypic sex matched, as provided by the UK Biobank. Sex was used as a covariate in all analyeses to remove the mean differences between males and females.
Reporting on race, ethnicity, or other socially relevant groupings	N/A
Population characteristics	Participants were between 40 and 69 years when they enrolled in the UK Biobank study between 2006 and 2010. Individuals were recruited from England, Wales and Scotland. The population consists of approximately 54% female participants. Further details are available from the UK Biobank manuscript: 10.1371/journal.pmed.1001779.
Recruitment	Invitations to participate in the UK Biobank were sent to 9.2M individuals registered in the UK's National Health Service who were 40-69 years old in 2006-2010 and lived within 40km of one of the 22 assessment centers located in England, Wales and Scotland. Further details are available from the UK Biobank manuscript: 10.1371/journal.pmed.1001779.
Ethics oversight	The UK Biobank study was approved by the North West Centre for Research Ethics Committee (11/NW/0382). Participants volunteered for the study and provided signed electronic consent on recruitment. Further details on the ethics and governance framework of the UK Biobank is available on the UK Biobank website [https://www.ukbiobank.ac.uk/] and from the UK Biobank manuscript: 10.1371/journal.pmed.1001779.

Note that full information on the approval of the study protocol must also be provided in the manuscript.

Field-specific reporting

Please select the one below that is the best fit for your research. If you are not sure, read the appropriate sections before making your selection.

☒ Life sciences ☐ Behavioural & social sciences ☐ Ecological, evolutionary & environmental sciences

Life sciences study design

All studies must disclose on these points even when the disclosure is negative.

Sample size	Data was obtained from the UK Biobank study and the authors were not involved in the data collection. We used the maximum number of individuals that were possible (125,000), given our computing resource limitations. For binary phenotypes, we always used all available cases to maximise power.
Data exclusions	UK Biobank (UKB) is a large prospective cohort study with genetic and health-related data collected on ~500,000 individuals aged between 40 and 69 years old living across the United Kingdom. The initial data processing steps of this dataset are described in detail in its original publications.<br>Given our computational resources and the likely availability of data from the PGS Catalog, the HRC+ UK10K imputed genome dataset’s variants were filtered to only keep HapMap3 SNPs, MAF > 0.001, maximum missingness per marker of 0.02, INFO > 0.9 that did not fail QC in any of the UKB batches. This process left 1,188,672 SNPs for further analyses. Samples were filtered to only keep unrelated (kinship coefficient < 0.0884), European ancestry individuals with concordant recorded and inferred sex. To accommodate the scale of analyses on the

available computing resources, this list of individuals was further filtered to 125,000 European ancestry individuals for each phenotype by keeping all cases (for binary traits) and prioritising to keep from the rest of the samples those with the most complete phenotype information.

Replication	Datasets were split in a pattern of 6:2:2 for training:validation:test sets. The test sets were held-out and not used in the training of the models. We have not attempted to replicate our results in external datasets as a similar sized dataset for replication was not available.
Randomization	Not applicable as our study was not experimental and there were no treatment groups.
Blinding	Not applicable as our study was not experimental and there were no treatment groups.

Reporting for specific materials, systems and methods

We require information from authors about some types of materials, experimental systems and methods used in many studies. Here, indicate whether each material, system or method listed is relevant to your study. If you are not sure if a list item applies to your research, read the appropriate section before selecting a response.

Materials & experimental systems

n/a	Involved in the study	
☒	☐	Antibodies
☒	☐	Eukaryotic cell lines
☒	☐	Palaeontology and archaeology
☒	☐	Animals and other organisms
☒	☐	Clinical data
☒	☐	Dual use research of concern
☒	☐	Plants

Methods

n/a	Involved in the study	
☒	☐	ChIP-seq
☒	☐	Flow cytometry
☒	☐	MRI-based neuroimaging

Plants

Seed stocks	*Report on the source of all seed stocks or other plant material used. If applicable, state the seed stock centre and catalogue number. If plant specimens were collected from the field, describe the collection location, date and sampling procedures.*
Novel plant genotypes	*Describe the methods by which all novel plant genotypes were produced. This includes those generated by transgenic approaches, gene editing, chemical/radiation-based mutagenesis and hybridization. For transgenic lines, describe the transformation method, the number of independent lines analyzed and the generation upon which experiments were performed. For gene-edited lines, describe the editor used, the endogenous sequence targeted for editing, the targeting guide RNA sequence (if applicable) and how the editor was applied.*
Authentication	*Describe any authentication procedures for each seed stock used or novel genotype generated. Describe any experiments used to assess the effect of a mutation and, where applicable, how potential secondary effects (e.g. second site T-DNA insertions, mosiacism, off-target gene editing) were examined.*

This checklist template is licensed under a Creative Commons Attribution 4.0 International License, which permits use, sharing, adaptation, distribution and reproduction in any medium or format, as long as you give appropriate credit to the original author(s) and the source, provide a link to the Creative Commons license, and indicate if changes were made. The images or other third party material in this article are included in the article's Creative Commons license, unless indicated otherwise in a credit line to the material. If material is not included in the article's Creative Commons license and your intended use is not permitted by statutory regulation or exceeds the permitted use, you will need to obtain permission directly from the copyright holder. To view a copy of this license, visit http://creativecommons.org/licenses/by/4.0/
